# Isolated Callosal Infarction Associated With Left Ventricular Thrombus in the Absence of Documented Atrial Fibrillation: A Case Report

**DOI:** 10.7759/cureus.100308

**Published:** 2025-12-29

**Authors:** Mark Jenzen H Trivilegio, Johnny K Lokin

**Affiliations:** 1 Neurology, University of Santo Tomas Hospital, Manila, PHL

**Keywords:** acute ischemic stroke (ais), cardioembolic stroke, cognitive impairment, corpus callosum infarction, left ventricular thrombus

## Abstract

Isolated corpus callosum infarction is an uncommon subtype of ischemic stroke and may be diagnostically challenging because of its atypical clinical presentation and variable clinicoradiologic correlation. We report the case of a 43-year-old Filipino male with poorly controlled hypertension who presented with sudden-onset right-sided weakness, expressive language impairment, and disorientation. On admission, the patient had a National Institutes of Health Stroke Scale score of 10. Neurologic examination revealed right central facial palsy, right hemiparesis, dysarthria, and cognitive impairment. Brain magnetic resonance imaging demonstrated acute ischemic lesions involving the body and splenium of the corpus callosum without definite cortical infarction. Extracranial vascular imaging showed no significant stenosis. Cardiac evaluation revealed a hypokinetic left ventricle with reduced ejection fraction and an apical thrombus, while continuous inpatient monitoring and 24-hour Holter electrocardiography did not demonstrate atrial fibrillation during hospitalization. The patient was managed with therapeutic anticoagulation, high-intensity statin therapy, optimization of antihypertensive treatment, and early multidisciplinary rehabilitation, resulting in meaningful neurological and functional improvement. This case illustrates the diagnostic complexity of callosal infarction and supports consideration of left ventricular thrombus as a clinically significant cardioembolic source, even when atrial fibrillation is not detected at initial presentation.

## Introduction

Isolated corpus callosum infarction is an uncommon subtype of ischemic stroke, accounting for approximately 1-3% of cases, a rarity largely attributed to the corpus callosum’s dual blood supply from the anterior and posterior cerebral arteries [1,2]. As the principal commissural fiber tract connecting the two cerebral hemispheres, lesions involving the corpus callosum often result in atypical and heterogeneous clinical manifestations rather than classic cortical stroke syndromes [2,3].

Clinically, corpus callosum infarction has been associated with interhemispheric disconnection syndromes, including gait disturbance, apraxia, agraphia, tactile anomia, and executive or behavioral dysfunction [3-6]. Motor and language deficits may occur, but are often subtle or disproportionate to the lesion location, making clinicoradiologic correlation challenging and potentially leading to a delayed diagnosis [5,6].

Although large-vessel disease and cardioembolism related to atrial fibrillation are the most common etiologies of corpus callosum infarction, less frequently recognized sources, such as left ventricular thrombus in patients without documented atrial fibrillation, have also been reported [7]. Recognition of these atypical embolic mechanisms is important in patients with uncommon clinical-radiologic stroke patterns, as illustrated in this case, where identification of a left ventricular thrombus provided a clinically significant cardioembolic explanation despite the absence of documented atrial fibrillation at the time of presentation.

## Case presentation

A 43-year-old right-handed Filipino male with a five-year history of poorly controlled hypertension presented to the emergency department with sudden-onset right-sided weakness, speech difficulty, and disorientation. The patient was last known well approximately 12 hours prior to admission, when he developed abrupt right upper and lower extremity weakness accompanied by slurred speech and confusion. He sought medical consultation shortly thereafter. There was no history of preceding trauma, seizure activity, headache, fever, or prior similar neurologic events. He had no known history of atrial fibrillation, ischemic heart disease, valvular disease, or prior stroke. He was a nonsmoker and reported occasional alcohol intake. Family history was noncontributory.

On admission, the patient had a National Institutes of Health Stroke Scale (NIHSS) score of 10, consistent with moderate stroke severity. Itemized deficits included impaired orientation (two points), right central facial palsy (two points), right arm weakness (one point), right leg weakness (three points), sensory impairment (one point), and dysarthria (one point). Given the 12-hour interval from symptom onset, the patient was outside the therapeutic window for intravenous recombinant tissue plasminogen activator (rTPA).

Neurologic examination revealed that the patient was awake but disoriented to time and place. Cranial nerve examination showed a right central facial palsy with mild dysarthria. Motor examination demonstrated right hemiparesis, more pronounced in the lower extremity than the upper extremity. Sensory examination revealed decreased sensation on the right side. Deep tendon reflexes were brisk on the right, with an extensor plantar response. No cortical signs were identified, including hemispatial neglect, visual field deficits, or gaze deviation. There were no cerebellar signs.

Language assessment demonstrated non-fluent verbal output with preserved comprehension and repetition, consistent with an expressive language deficit. Cognitive evaluation revealed impairments in attention, executive function, and visuospatial processing, accompanied by psychomotor slowing and reduced verbal initiative.

**Figure 1:**
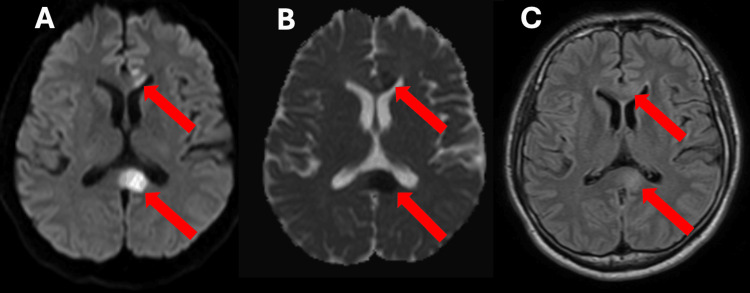
Cranial MRI findings (A) Diffusion-weighted imaging (DWI) shows areas of hyperintensity involving the body and splenium of the corpus callosum (red arrows), consistent with restricted diffusion. (B) Apparent diffusion coefficient (ADC) map demonstrates corresponding hypointense signals in the same regions (red arrows), demonstrating true diffusion restriction. (C) Fluid-attenuated inversion recovery (FLAIR) sequence shows subtle hyperintensity in the body and splenium of the corpus callosum (red arrows), corresponding to the areas of restricted diffusion.

Baseline laboratory investigations, including complete blood count, serum electrolytes, renal and liver function tests, fasting lipid profile, and glycosylated hemoglobin, were within normal limits. Brain magnetic resonance imaging (MRI) with diffusion-weighted imaging (DWI) demonstrated acute ischemic infarcts involving the body and splenium of the corpus callosum, without evidence of adjacent cortical or subcortical involvement. Apparent diffusion coefficient (ADC) sequences showed corresponding hypointensity, and fluid-attenuated inversion recovery (FLAIR) images demonstrated subtle signal changes consistent with a hyperacute infarct (Figure 1).

**Figure 2:**
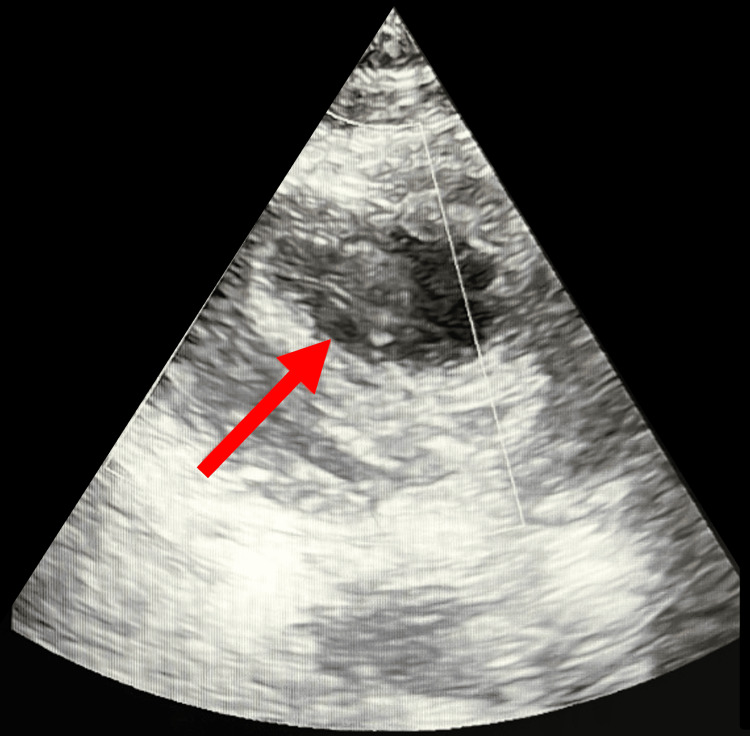
Transthoracic echocardiogram Two-dimensional transthoracic echocardiography demonstrating a homogeneous, echo-dense mass adherent to the anterolateral apical wall of the left ventricle (red arrow), consistent with a left ventricular thrombus in the setting of reduced left ventricular systolic function.

Extracranial vascular evaluation using carotid duplex ultrasonography revealed less than 50% stenosis of the bilateral internal and external carotid arteries, excluding significant extracranial atherosclerotic disease. In view of the absence of a clear arterial source, a cardioembolic etiology was pursued. Continuous inpatient cardiac monitoring and 24-hour Holter electrocardiography did not demonstrate atrial fibrillation during hospitalization. Transthoracic echocardiography revealed a hypokinetic left ventricle with a reduced ejection fraction of 39% and a homogeneous, echo-dense thrombus adherent to the anterolateral apical wall, consistent with a left ventricular thrombus (Figure 2).

Based on the clinical presentation, neuroimaging findings, and cardiac evaluation, the patient was diagnosed with acute ischemic stroke involving the corpus callosum, most likely secondary to cardioembolism from a left ventricular thrombus.

Therapeutic anticoagulation was initiated with subcutaneous enoxaparin 60 mg (0.6 mL) administered twice daily, followed by transition to oral apixaban 5 mg twice daily for long-term secondary stroke prevention. High-intensity statin therapy was started with atorvastatin 80 mg once daily. Antihypertensive treatment was optimized using a calcium channel blocker and an angiotensin-converting enzyme inhibitor, titrated to achieve blood pressure control. Early multidisciplinary rehabilitation was instituted, including physical, occupational, and cognitive therapy. The patient demonstrated gradual neurological improvement during hospitalization, with improvement in motor strength, speech output, and functional independence at the time of discharge.

## Discussion

The corpus callosum is the principal interhemispheric commissural pathway, facilitating integration of motor, sensory, and higher cognitive functions between the cerebral hemispheres. Owing to its dual vascular supply from branches of the anterior and posterior cerebral arteries, isolated infarction of the corpus callosum is uncommon, accounting for less than 3% of ischemic strokes [1,2]. When present, callosal infarction often produces atypical and heterogeneous clinical manifestations, frequently lacking classic cortical signs and posing diagnostic challenges [3-6].

In this case, neuroimaging demonstrated acute ischemic lesions confined to the body and splenium of the corpus callosum, a pattern consistent with previously reported cases of isolated callosal infarction [2,3]. Clinically, callosal strokes have been associated with interhemispheric disconnection syndromes, executive dysfunction, apraxia, behavioral changes, and, less commonly, motor or language disturbances [4-6]. The patient’s presentation with right-sided hemiparesis and expressive language impairment demonstrates the complex clinicoradiologic correlation seen in callosal infarction. Although dense hemiparesis is uncommon in isolated callosal lesions, disruption of interhemispheric motor integration may result in apparent unilateral weakness despite preservation of the primary motor cortex [5,6].

An additional consideration is the possibility of subradiographic cortical ischemia, particularly in cardioembolic stroke. Prior studies have demonstrated that a small proportion of acute ischemic strokes, estimated at approximately 2%, may not be detected on initial MRI, especially when lesions are small, cortical, or very early in evolution [6]. In this patient, the presence of a left ventricular thrombus provides a biologically plausible embolic source capable of producing multiple small emboli, potentially accounting for clinical deficits that appear disproportionate to visible imaging findings. A follow-up MRI would be useful to assess for the evolution of cortical infarcts or progression of the callosal lesion.

Reversible splenial lesion syndrome (RESLES) was also considered as a differential diagnosis, given the involvement of the splenium of the corpus callosum. RESLES is a clinicoradiological entity characterized by transient callosal lesions associated with metabolic disturbances, seizures, infections, or toxic exposures, and typically resolves within weeks without specific treatment [3]. However, in this case, the presence of focal neurologic deficits, diffusion restriction involving both the body and splenium of the corpus callosum, vascular risk factors, and identification of a definite cardioembolic source favor an ischemic mechanism rather than a reversible metabolic or toxic process. Nonetheless, the absence of follow-up imaging represents a limitation, as resolution of the callosal lesion on repeat MRI would support the diagnosis of RESLES.

From a therapeutic standpoint, this case shows the importance of identifying uncommon cardioembolic sources. Left ventricular thrombus is a recognized but often underappreciated cause of ischemic stroke, even in the absence of atrial fibrillation [7]. Early initiation of therapeutic anticoagulation is essential to reduce the risk of recurrent embolic events. In this patient, anticoagulation with low-molecular-weight heparin followed by transition to a direct oral anticoagulant, combined with high-intensity statin therapy, optimization of blood pressure control, and early multidisciplinary rehabilitation, resulted in meaningful neurological and functional improvement. These management strategies are consistent with available evidence supporting anticoagulation for left ventricular thrombus and comprehensive secondary stroke prevention [8].

## Conclusions

This case describes an uncommon presentation of isolated corpus callosum infarction with atypical clinical features and variable clinicoradiologic correlation. The presence of motor weakness and expressive language impairment suggests a clinicoradiologic mismatch, which may reflect interhemispheric disconnection and/or concurrent subradiographic cortical ischemia not visualized on initial imaging. Identification of a left ventricular thrombus supports consideration of a cardioembolic mechanism, even when atrial fibrillation is not detected during hospitalization, recognizing that paroxysmal atrial fibrillation may require extended monitoring for detection. Timely initiation of anticoagulation, together with comprehensive secondary stroke prevention and early rehabilitation, was associated with meaningful neurological and functional improvement. Follow-up neuroimaging and prolonged cardiac rhythm monitoring may further enhance diagnostic accuracy and guide long-term management in similar presentations.
